# The Concentration of Iron in Real-World Geogenic PM_10_ Is Associated with Increased Inflammation and Deficits in Lung Function in Mice

**DOI:** 10.1371/journal.pone.0090609

**Published:** 2014-02-28

**Authors:** Graeme R. Zosky, Thomas Iosifidis, Kara Perks, Will G. F. Ditcham, Sunalene G. Devadason, W. Shan Siah, Brian Devine, Fiona Maley, Angus Cook

**Affiliations:** 1 School of Medicine, University of Tasmania, Hobart, Tasmania, Australia; 2 Cooperative Research Centre for Asthma and Airways, Sydney, New South Wales, Australia; 3 Telethon Institute for Child Health Research, Subiaco, Western Australia, Australia; 4 School of Paediatrics and Child Health, The University of Western Australia, Subiaco, Western Australia, Australia; 5 School of Population Health, The University of Western Australia, Crawley, Western Australia, Australia; University of Tennessee Health Science Center, United States of America

## Abstract

**Background:**

There are many communities around the world that are exposed to high levels of particulate matter <10 µm (PM_10_) of geogenic (earth derived) origin. Mineral dusts in the occupational setting are associated with poor lung health, however very little is known about the impact of heterogeneous community derived particles. We have preliminary evidence to suggest that the concentration of iron (Fe) may be associated with the lung inflammatory response to geogenic PM_10_. We aimed to determine which physico-chemical characteristics of community sampled geogenic PM_10_ are associated with adverse lung responses.

**Methods:**

We collected geogenic PM_10_ from four towns in the arid regions of Western Australia. Adult female BALB/c mice were exposed to 100 µg of particles and assessed for inflammatory and lung function responses 6 hours, 24 hours and 7 days post-exposure. We assessed the physico-chemical characteristics of the particles and correlated these with lung outcomes in the mice using principal components analysis and multivariate linear regression.

**Results:**

Geogenic particles induced an acute inflammatory response that peaked 6 hours post-exposure and a deficit in lung mechanics 7 days post-exposure. This deficit in lung mechanics was positively associated with the concentration of Fe and particle size variability and inversely associated with the concentration of Si.

**Conclusions:**

The lung response to geogenic PM_10_ is complex and highly dependent on the physico-chemical characteristics of the particles. In particular, the concentration of Fe in the particles may be a key indicator of the potential population health consequences for inhaling geogenic PM_10_.

## Introduction

The relative contribution of geogenic (earth derived) dusts to the total suspended particulates (TSPs) varies geographically. In some areas, such as urban centres, these dusts typically represent only a minor proportion of TSPs [1], [2] whereas in other regions, notably those prone to aridity and high levels of wind erosion, they may predominate [1], [3]. Importantly, the physico-chemical characteristics of suspended geogenic dusts in a particular area will vary depending on local geology and anthropogenic activities [4]. As such, the respiratory health implications for inhaling geogenic dusts are also likely to vary spatially.

It is well-established that mineral dusts in the occupational setting have pro-inflammatory properties [5], [6], [7], [8]. However, much less is known about the effect of inhaled geogenic particles on lung health when exposure occurs at a community level. Such dusts are likely to be physically and chemically heterogeneous, in contrast to dusts in the occupational setting which are usually consistent in size and chemical composition. We have previously shown that community sampled geogenic particles less than 10 µm in aero-dynamic diameter (PM_10_) have the capacity to induce a dose-dependent inflammatory response in the lung [9]. Importantly, the response varied between particles collected from the two sites that we studied. The most striking physico-chemical difference between the particle sources was the concentration of iron (Fe) with a higher concentration of Fe being associated with a greater influx of neutrophils and a higher production of pro-inflammatory cytokines.

While much of the research in this field has focused on the lung response to inhaled silica (Si) particles due to their importance in occupational lung disease, there is recognition that other metals can have adverse impacts on the lung when inhaled; including Fe. Iron homeostasis in the lung is tightly regulated due to its importance in cell function and host defence [10]. The application of exogenous iron to the system can result in the production of oxidative stress [11] and synergistically enhance the response to other particles [12]. Exposure to dust from iron oxide stockpiles has also been associated with increased hospitalisations for respiratory disease [13] and in particular increased respiratory infections in children [14].

While our previous study [9] anecdotally suggests that Fe may influence the magnitude of the response in heterogeneous particles the fact that we only used two particle sources and did not have measures of lung function limited our capacity to probe this observation. In this study we aimed to use real-world community sampled geogenic PM_10_ to assess the impact of the association between the concentration of Fe, after controlling for other particle characteristics, and the lung response using an established mouse model.

## Methods

### Animals

Eight-week old females BALB/c mice were purchased from the Animal Resource Centre (ARC, Murdoch, Western Australia) and housed at the Telethon Institute for Child Health Research under a 12∶12 hour light:dark cycle. Food and water were provided *ad libitum*. All experiments were approved by the Telethon Institute Animal Ethics Committee (AEC #249) and conformed to the guidelines of the National Health and Medical Research Council of Australia.

### Geogenic Particle Extraction (PM_10_)

Surface soil samples were collected from four towns throughout the arid regions of Western Australia (Table 1). No specific permissions were required for access to these sites or for these collection activities. At each town, two samples from areas exposed to wind erosion and lacking vegetation were collected and processed using an established technique that allows extraction of PM_10_ at sufficient quantities for *in vivo* [15]. We have previously demonstrated that the chemical composition of PM_10_ obtained using this technique correlates strongly with airborne particles [9]. In this particular study we were not seeking to assign specific health risks to particular towns. Rather we sought to obtain PM_10_ samples with varying physico-chemical characteristics in order to establish how variation in these characteristics impacts on the lung response.

**Table 1:** Geographical location and estimated populations of permanent residents of the towns where the geogenic PM_10_ was sampled.

Town	Site	Location	Population
Newman	1	23°22′09″ S 119°43′58″ E	4245 (∼8000)
	2	23°21′25″ S 119°43′51″ E	
Tom Price	1	22°36′25″ S 117°56′10″ E	2721 (∼4000)
	2	22°40′56″ S 117°47′45″ E	
Kalgoorlie	1	30°43′52″ S 121°27′21′ E	31107
	2	30°45′45″ S 121°27′24′ E	
Karratha	1	20°43′46′ S 116°50′53″ E	11700 (∼15000)
	2	20°44′06′ S 116°48′18″ E	

The numbers in parentheses indicate the total population when the itinerant population of mine workers is included.

Two of the towns, Newman and Tom Price, are closely aligned with open cut mining for iron ore and have itinerant populations. Kalgoorlie has a stable population and is situated near open cut gold mining activities while Karratha is a coastal iron ore port. All of these towns are exposed to high levels of particulate matter that are largely geogenic in origin.

### Particle Characterisation

Endotoxin levels were assessed using a limulus amebocyte lysate (LAL) assay (GenScript; New Jersey, U.S.A.). For this purpose samples were diluted 1∶1000, adjusted for pH (6–8) and incubated in LAL at 37°C for 10 minutes. A chromogenic substrate was added and the absorbance was read at 405 nm and compared to an *E.coli* standard.

The chemical composition for a panel of 12 common metals (aluminium, Al; iron, Fe; cadmium, Cd; cobalt, Co; chromium, Cr; copper, Cu; arsenic, As; manganese, Mn; nickel, Ni; lead, Pb; uranium, U; zinc, Zn) was assessed by ICP-MS (Chem Centre, Western Australia). Samples were prepared according to the USEPA 3051A Method using acid digestion. The quantity of silica (SiO_2_) in the samples was assessed by ICP-OES (University of Western Australia) which was used to estimate the amount of Si in the particles.

Particle size was assessed using an Andersen Cascade Impactor (Copley Scientific, UK) using a previously described technique [9]. The mass median aerodynamic diameter (MMAD) and geometric standard deviation (GSD) of the particle sizes were calculated using an online tool (http://mmadcalculator.com/andersen-impactor-mmad.html).

### Exposure Protocol

Mice (n = 13 per group) were intranasally exposed to 100 µg of geogenic particles suspended in 50 µL of saline (+0.5% Tween-80) or vehicle (saline +0.5% Tween-80) alone under light methoxyflurane anaesthesia. Mice were studied for the outcomes (described below) at 6 hours, 24 hours or 7 days post-exposure. The limited quantity of PM_10_ available prevented exposure by dry aerosol. We chose to use intranasal exposure over intratracheal instillation to avoid a biased deposition of the particles in the large airways [16] and to ensure that the particles were able to reach the alveoli we used an optimised protocol with a delivery volume of 50 µL and mild sedation [17]. The 100 µg dose was chosen based on the dose-response data from our previous study [9] as a dose that produces a robust and measurable inflammatory response. This dose (∼5 mg.kg^−1^) is an order of magnitude smaller than typical rodent particle exposure models (∼100 mg.kg^−1^ [18]) and is consistent with our prior work on diesel exhaust particles [19], [20].

### Lung Mechanics and Lung Volume

Mice were prepared for *in vivo* assessment of lung function as described previously [21]. Following anaesthesia, surgical tracheostomy and standardisation of lung volume history [21] baseline lung volume at 0 cmH_2_O transrespiratory pressure (P_rs_) was determined [22]. Briefly, thoracic gas volume (TGV) at 0 cmH_2_O P_rs_ (V_0_) was calculated by the relationship between tracheal pressure and plethysmograph (box) pressure following electrical stimulation of the inspiratory muscles [22].

Lung mechanics were then measured using a modification of the forced oscillation technique. During pauses in ventilation, an oscillatory signal containing 9 frequencies (4–38 Hz) was generated by a loudspeaker and passed through a wavetube to the mouse via the tracheal cannula. The respiratory system impedance (Z_rs_) was measured as the load impedance on the wavetube. A four parameter model with constant phase tissue impedance was fitted to the Z_rs_ data [23] to obtain measures of R_aw_, the Newtonian resistance which is equivalent to airway resistance in the mouse due to the compliance of the chest wall, G (tissue damping) which is thought to represent the resistance of the small airways where air movement occurs primarily by diffusion and H (tissue elastance) the stiffness of the lung parenchyma. Hysteresivity (η = G/H), was calculated as a marker of lung heterogeneity [24]. Following the measurement of baseline mechanics a further TGV measurement was made prior to a slow inflation deflation manoeuvre from 0 to 20 cmH_2_O P_rs_. By integrating flow through the wavetube we estimated the maximum lung volume achieved at 20 cmH_2_O P_rs_ (V_20_) as an index of lung capacity [25].

### Inflammatory Markers

Following the measurement of lung function, mice were euthanased by anaesthetic overdose. A bronchoalveolar lavage (BAL) was obtained as described previously [9] in order to assess inflammatory cells and cytokines. The concentrations of IL-6, MIP-2 (mouse IL-8 analogue) and IL-1β in the BAL were determined by ELISA according to the manufacturer’s instructions (MIP-2, R&D Systems, Minnesota, U.S.A; IL-6, IL-1β, MCP-1, BD Biosciences, California, U.S.A.) from the lavage supernatant.

### Statistical Analyses

In order to deal with the volume of data generated in this study we took a hierarchical approach to the analysis. Firstly, we compared the response for each outcome (within each time point) to the saline group (ANOVA following by Holm-Sidak post-hoc tests with the saline group as the control comparison). We used this analysis to identify which outcomes were altered by exposure to geogenic PM_10_.

In this study we were interested in exploring the key characteristics of the geogenic particles that were associated with these specific lung outcomes. In order to explore these associations a two stage analysis was conducted. Firstly, we conducted a principal components factor analysis of the lung outcomes that were identified in the first ANOVA as being significantly altered by exposure to geogenic PM_10_ (macrophages, neutrophils, IL-6, MIP-2, IL-1β, G, H, η). In order to satisfy the assumption of normal distribution of the error terms the data were transformed where appropriate using Box-Cox or logarithmic transformations. We assessed the screeplot to determine the number of factors to include and used orthogonal rotation. The results of this factor analysis were then used to create “scores” for each mouse. These scores were used in multivariate linear regression analyses at each time using the concentrations of Fe, Si, Al (i.e. metals with >0.015% representation in each sample), endotoxin, MMAD and GSD as variables.

## Results

### Particle Characteristics

The physico-chemical characteristics of the particles varied considerably between sites (Table 2). The particles were dominated by Si, Al and Fe. Endotoxin levels varied considerably between samples but were generally low with the highest concentration (∼158 EU/50 µL) equivalent to approximately 16 ng in the exposure solution.

**Table 2:** Summary of the physico-chemical characteristics of the geogenic PM_10_ from each of the sites.

	*Newman*		*Tom Price*		*Kalgoorlie*		*Karratha*	
	1	2	1	2	1	2	1	2
*MMAD (µm)*	4.5	2.93	2.45	4.27	1.52	3.45	3.42	2.68
*GSD*	2.06	2.49	2.24	2.56	4.65	3.4	4.95	2.63
*Si (mg.kg* ^−*1*^ *)*	188000	186000	182000	188000	175000	170000	213000	191000
*Al (mg.kg* ^−*1*^ *)*	75100	84000	58600	70600	85300	82200	70400	70800
*Fe (mg.kg* ^−*1*^ *)*	95000	120000	120000	100000	62000	60000	82000	93000
*Mn (mg.kg* ^−*1*^ *)*	970	1600	1300	1500	870	860	1200	860
*Cr (mg.kg* ^−*1*^ *)*	190	210	81	200	340	360	210	250
*Zn (mg.kg* ^−*1*^ *)*	150	100	73	180	200	85	100	76
*Ni (mg.kg* ^−*1*^ *)*	140	110	43	110	150	140	100	87
*Cu (mg.kg* ^−*1*^ *)*	83	97	57	120	74	47	100	95
*Pb (mg.kg* ^−*1*^ *)*	33	49	27	26	47	2100	42	70
*Co (mg.kg* ^−*1*^ *)*	43	52	39	56	36	24	33	28
*As mg.kg* ^−*1*^ *)*	12	8	11	15	19	34	7	12
*U (mg.kg* ^−*1*^ *)*	0.78	1	1.6	0.66	1.3	0.91	0.61	0.93
*Cd (mg.kg* ^−*1*^ *)*	0.54	0.05	0.05	0.29	0.3	0.08	0.05	0.05
*Endo (ng/50 µL)*	11.5	0.5	0.5	15.8	4.2	2.3	0.5	3.1

Two independent samples were collected at each site.

### Responses Compared to Control (Saline) Mice

**Figure 1:**
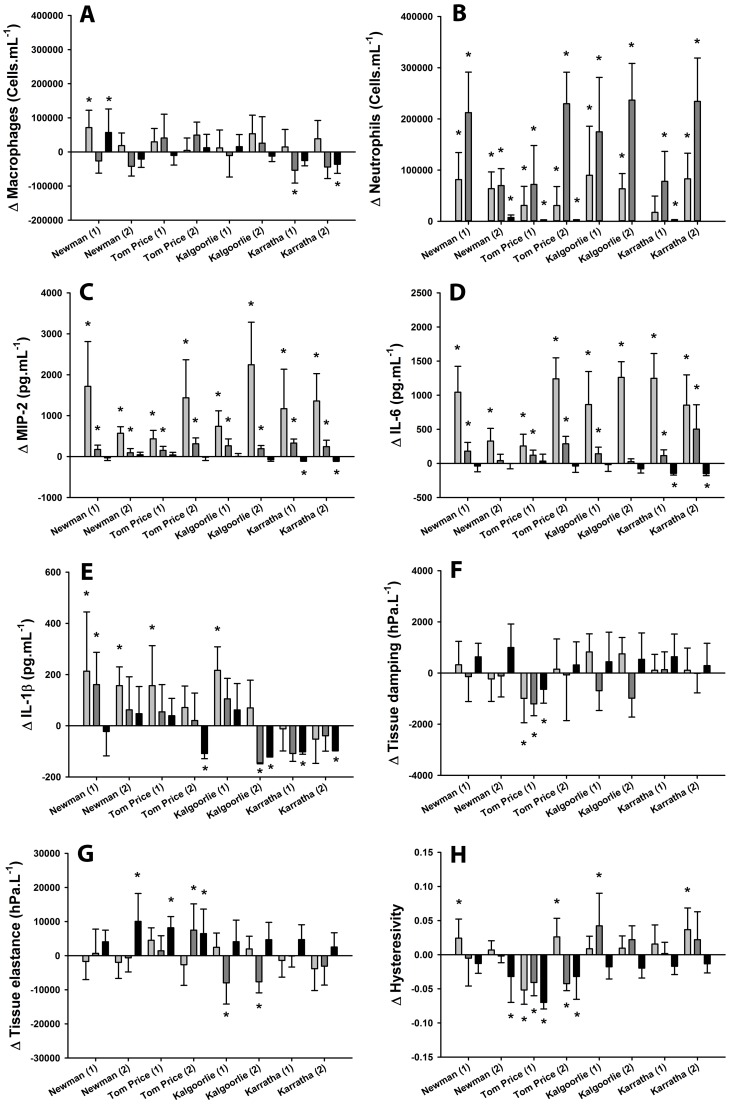
The inflammatory and lung function response to geogenic particles in mice varies depending on the origin of the particles. Levels of macrophages (A), neutrophils (B), MIP-2 (C), IL-6 (D), IL-1β (E), tissue damping (F), tissue elastance (G) and hysteresivity (H) in mice 6 hours (light grey bars), 24 hours (dark grey bars) and 7 days (black bars) post-exposure to geogenic PM_10_. Data are mean(SD) and are expressed relative to the mean response in saline (control) exposed mice. *indicates p<0.05 compared to saline at the equivalent timepoint.

Data for control mice are presented in Table 3. The lung response to exposure to 100 µg of geogenic PM_10_ was highly variable (Figure 1). Exposure to geogenic PM_10_ caused a significant influx of neutrophils that peaked 24 hours post exposure and had returned to control levels at the 7 day timepoint (Figure 1). There was also a macrophage response, although the pattern was less consistent. There was a concomitant increase in inflammatory cytokines which, in the case of MIP-2 and IL-6, peaked 6 hours post-exposure, was still evident 24 hours post-exposure and had returned to control levels by the 7 day timepoint. Interestingly, in some cases, the levels of inflammatory cytokines were below control levels at the later timepoints. There was a similar pattern in IL-1β, although this response was more variable (Figure 1). We did not detect significant levels of MCP-1 in the BAL.

**Table 3:** Results for control (saline exposed) mice for the outcome measures of interest 6 hours, 24 hours and 7 days post-exposure.

Outcome	Time post-exposure		
	*6 hours*	*24 hours*	*7 days*
Macrophages (Cells.mL^−1^)	58.4×10^3^ (25.1×10^3^)	104.7×10^3^ (36.7×10^3^)	79.6×10^3^ (39.3×10^3^)
Neutrophils (Cells.mL^−1^)	1.0×10^3^ (1.3×10^3^)	1.1×10^3^ (1.2×10^3^)	0.7×10^3^ (1.0×10^3^)
MIP-2 (pg.mL^−1^)	172.3 (65.1)	142.0 (48.0)	181.6 (128.2)
IL-6 (pg.mL^−1^)	129.1 (48.7)	112.0 (70.5)	197.8 (155.0)
IL-1β (pg.mL^−1^)	213.1 (108.0)	219.1 (169.3)	192.5 (115.9)
Tissue damping (hPa.L^−1^)	7550 (540)	8020 (1200)	7120 (920)
Tissue elastance (hPa.L^−1^)	34200 (2800)	36700 (7100)	30600 (6200)
Hysteresivity	0.22 (0.01)	0.22 (0.01)	0.24 (0.03)

Data are mean(SD).

Responses in lung mechanics were highly variable between groups. Exposure to particles from Tom Price “1” caused a decrease (“improvement”) in tissue damping at all timepoints, while tissue elastance increased (“deficit”) 7 days post exposure in the same mice. Exposure to particles from Newman “2” and Tom Price “2” caused an increase in tissue elastance at the later timepoints, whereas exposure to particles from Kalgoorlie appeared to improve tissue elastance (Figure 1). Hysteresivity varied between exposure groups (Figure 1). There was no evidence for an effect of exposure to these particles on R_aw_, V_0_ or V_20_ (*data not shown*).

### Factor Analysis

Based on the results of the previous analyses the following variables were included in the principal components analysis: neutrophils, macrophages, MIP-2, IL-6, IL-1β, tissue damping, tissue elastance and hysteresivity. Examination of the screeplot revealed a distinct break point after two factors (∼58.9% of the variance explained). Inclusion of additional factors only resulted in incremental improvements in the explained variance. The first factor that we identified was strongly (|>0.75|) loaded on (+) MIP-2 and (+) IL-6 and moderately (|>0.50|) loaded on (+) neutrophils and (+) IL-1β (Table 4). As such, this factor described the inflammatory response to the geogenic PM_10_. The second factor we identified was strongly (|>0.75|) loaded on (+) tissue elastance and moderately (|>0.50|) loaded on (+) tissue damping and (–) hysteresivity. As such, this factor described the lung function response to the geogenic PM_10_.

**Table 4:** Results of the principal components factor analysis for the eight outcomes included in model, with loadings for each outcome.

Outcome	Factor 1	Factor 2
Macrophages	0.38	0.00
Neutrophils	*0.52*	−0.47
MIP-2	**0.90**	−0.18
IL-6	**0.90**	−0.18
IL-1β	*0.69*	0.18
Tissue damping	0.09	*0.58*
Tissue elastance	−0.12	**0.97**
Hysteresivity	0.28	−*0.71*

**Figure 2:**
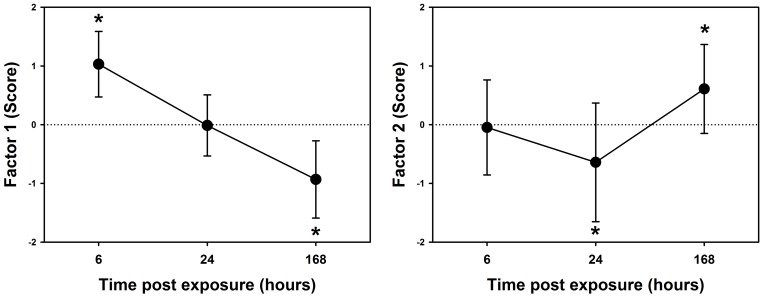
The lung response to geogenic particles was characterised by acute (6 hours post-exposure) inflammation and long term (7 days post-exposure) deficits in lung function. Mean(SD) scores for Factor 1 (inflammation, left) and Factor 2 (lung function, right) for all mice exposed to geogenic PM_10_ obtained following principal components factor analysis. *indicated p<0.05 against zero.

Using these factors we generated scores for each mouse. Pooling these scores across sites revealed the following patterns; 1) the general inflammatory response peaked 6 hours post-exposure but then (on average) fell below control levels 7 days post-exposure, and 2) the lung function response showed evidence of a paradoxical “improvement” in lung mechanics 24 hours post-exposure followed by a deficit 7 days post-exposure (Figure 2).

### Physico-chemical Characteristics and the Lung Response

We used the four primary responses we identified in the factor analysis (1: acute inflammatory response 6 hours post-exposure, 2: decrease in inflammatory markers 7 days post-exposure below control levels, 3: “improvement” in lung mechanics 24 hours post-exposure, 4: deficit in lung mechanics 7 days post-exposure) in order to identify which physico-chemical characteristics of the geogenic PM_10_ were associated with each lung response. These relationships were assessed using multivariate linear regression with the Fe, Si, Al (the most abundant metals), MMAD, GSD and endotoxin levels as the independent variables (Table 5).

**Table 5:** Results of the multi-variate linear regression analysis examining the association between the physico-chemical characteristics of the geogenic PM_10_ and the primary lung responses identified in the factor analysis.

	Factor 1 (Inflammation)				Factor 2 (Lung function)			
	*6 hours post-exposure*		*7 days post-exposure*		*24 hours post-exposure*		*7 days post-exposure*	
	[95% CI]		[95% CI]		[95% CI]		[95% CI]	
Fe (mg.kg^−1^)	−0.014	**0.007**	0.041	**<0.001**	0.034	**<0.001**	0.029	**<0.001**
	[−0.025, −0.004]		[0.031, 0.050]		[0.018, 0.051]		[0.013, 0.044]	
Si (mg.kg^−1^)	−0.006	0.47	−0.047	**<0.001**	−0.009	0.52	−0.031	**0.01**
	[−0.022, 0.010]		[−0.062, −0.031]		[−0.035, 0.018]		[−0.056, −0.006]	
Al (mg.kg^−1^)	0.001	0.13	0.018	**0.01**	−0.020	0.10	0.008	0.47
	[−0.003, 0.026]		[0.004, 0.032]		[−0.044, 0.004]		[−0.014, 0.031]	
MMAD (µm)	0.147	0.13	0.084	0.36	0.162	0.30	0.159	0.28
	[−0.044, 0.339]		[−0.100, 0.267]		[−0.150, 0.475]		[−0.131, 0.450]	
GSD	−0.146	0.26	0.618	**<0.001**	0.402	0.06	0.443	**0.03**
	[−0.402, 0.110]		[0.372, 0.864]		[−0.016, 0.820]		[0.054, 0.833]	
Endo (ng)	0.003	0.80	0.033	**0.007**	0.081	**<0.001**	0.001	0.98
	[−0.021, 0.027]		[0.009, 0.056]		[0.039, 0.123]		[−0.036, 0.037]	

We found that the concentration of Fe was the only characteristic associated (p = 0.007) with the early inflammatory response. This was an inverse relationship such that as the concentration of iron increased, the magnitude of this response decreased. In contrast, the decrease in inflammatory markers below control levels we observed 7 days post-exposure was associated with a number of characteristics including (–) Si [p<0.001], (+) Al [p = 0.01], (+) Fe [p<0.001], (+) GSD [p<0.001] and (+) endotoxin levels [p = 0.007]. It is important to note that a positive association in this instance indicates that as the value of the predictor *increased* the magnitude of the deficit in inflammatory markers below control levels *decreased*. In other words, an increase in the concentration of Si was associated with a bigger “deficit” in inflammatory cytokines compared to control mice 7 days post-exposure.

The apparent “improvement” in lung mechanics 24 hours post-exposure was associated with (+) Fe [p<0.001] and (+) endotoxin levels [p<0.001]. In this case, the sign of these associations indicates that as both the concentration of Fe and endotoxin in the geogenic PM_10_ increased, the magnitude of the improvement in lung mechanics *decreased*.

The long term deficit in lung mechanics we observed 7 days post-exposure was associated with (–) Si [p = 0.01], (+) Fe [p<0.001] and (+) GSD [p = 0.03]. These associations have a straightforward interpretation such that as the concentration of Fe and the variability in size of the particles (GSD) increased, the magnitude of the deficit in lung mechanics also increased. In contrast, this deficit was inversely associated with the concentration of Si.

## Discussion

The magnitude and pattern of the lung response to instilled geogenic PM_10_ in mice varied considerably depending on the source of the particles. The response was characterised by 1) an acute inflammatory response including the production of pro-inflammatory cytokines, such as MIP-2 and IL-6 and an influx of neutrophils, 2) a reduction in inflammatory markers 7 days post-exposure below control levels and, 3) an apparent improvement in lung mechanics 24 post-exposure, which was followed by a deficit in lung mechanics 7 days post-exposure. Increasing concentrations of Fe in the samples were inversely associated with the acute inflammatory response, but were positively associated with a reduction in the deficit in inflammatory markers and an increase in the lung impairment observed 7 days-post exposure. A similar pattern was observed for the variability in particle size (GSD). Conversely, higher concentrations of Si were associated with a greater reduction in the inflammatory response below control levels and a smaller impairment in lung mechanics 7 days post-exposure.

We have previously shown that community-sampled geogenic PM_10_ induces a potent inflammatory response in the lung [9]. This neutrophil dominated response is consistent with many previous studies examining the lung response to inhaled dusts (primarily SiO_2_) of mineral origin [18], [26]. While exposure to crystalline silica in mouse models elicits fibrotic changes [27], [28], which presumably alters lung function, there appears to be very little data in the literature on the impact of silica on lung function in mouse models (although silicosis in humans is associated with impaired lung function [29]). It should be noted that the exposure doses used in our study (∼5 mg.kg^−1^) are an order of magnitude lower than the doses typically used in mouse studies of pure crystalline silica exposure (e.g. ∼100 mg.kg^−1^, [18]) and silica only constituted a proportion of our particles. These observations suggest that the lung was particularly sensitive to the heterogeneous geogenic particulates used in our study.

The potent neutrophilia we observed has been linked to structural degradation of lung tissue in a variety of contexts [30], [31], [32] including exposure to mineral particles [33]. This neutrophil response was preceded by the production of pro-inflammatory cytokines. In addition to the potential for direct damage to the lung tissue resulting from the production of these cytokines, this response may also have important implications for an individual’s response to a secondary insult such as a bacterial [34] or viral lung infection [20].

As discussed earlier, there is a substantial body of work on the influence of silica based particles on the lung due to their importance in occupational lung disease. Given the dominance of Si in our samples (in the form of SiO_2_), it is likely that this mineral was a key driver of the primary response to the particles. Interestingly, an increase in the concentration of Si in our samples was associated with an increase in the magnitude of the deficit in inflammatory markers 7 days post-exposure (below control levels) yet a *decrease* in the magnitude of the deficit in lung mechanics we observed at the same timepoint. Given the large body of evidence showing a positive association between silica and poor lung outcomes this was a surprising result. Absolute levels of SiO_2_ in ambient particulates may therefore be a poor marker of the risk posed by geogenic particles to lung health.

**Figure 3:**
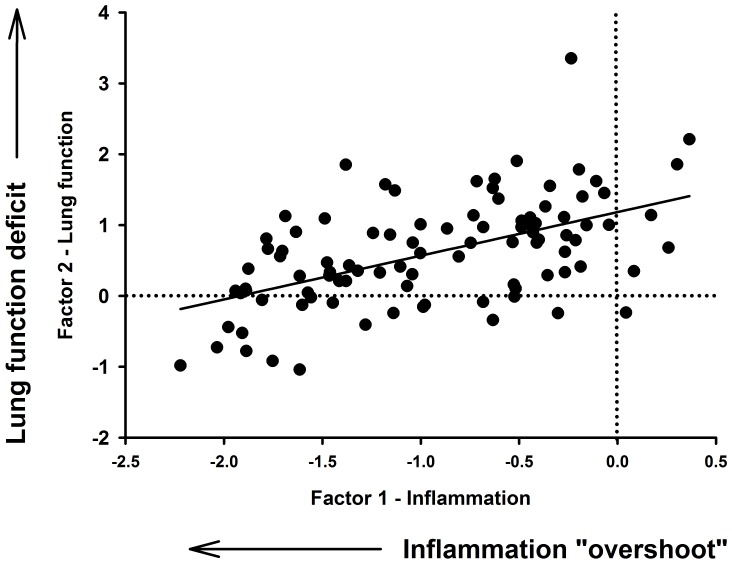
Deficits in lung function in response to inflammation were associated with corresponding decreases in inflammatory markers below baseline levels. Scatter plot showing the correlation (r = 0.54) between Factor 2 (lung function) and Factor 1 (inflammation) from mice 7 days post-exposure to geogenic PM_10_. Mice with greater declines in inflammatory markers (below control levels) 7 days post-exposure tended to have better lung function.

The only metal that was associated with all of the responses we observed was iron. The direction of the association between the responses we observed 7 days post-exposure and Fe was the opposite of that observed for Si: that is, elevated concentrations of Fe were linked to increases in the magnitude of the deficit in lung mechanics. Paradoxically, an increase in the concentration of Fe was associated with a smaller inflammatory response 6 hours post-exposure. This observation, combined with the link between the 7 day response we also found in the Si analysis, implies that the initial inflammatory response may not be the primary determinant of the long term deficit in lung mechanics. We confirmed this association by examining the correlation between the two “factors” we identified at the 7 day timepoint (Figure 3). This appears to indicate that the degree of “overshoot” of the inflammatory response 7 days post-exposure is inversely associated with the deficit in lung mechanics at that time. We postulate that this may reflect the lung attempting to “protect” itself from the adverse effect of over production of pro-inflammatory cytokines. In situations where this inflammatory “overshoot” is not triggered, for example when the concentration of Fe in the particles is high, then there were long lasting impacts on lung function as a consequence. This observation implies that it is the sustained production of cytokines, rather than the magnitude of the initial response, that is the key driver of the lung function impairments associated with exposure to geogenic PM_10_.

Iron is well known for its role in modulating responses to additional lung insults [35] and local Fe levels are tightly regulated to maintain normal physiological function [10]. However, the direct impact of iron particles on lung inflammation is equivocal, with some studies showing no effect [12], [36] and others showing some potentially detrimental responses [37]. It is possible that the negative response induced by Fe requires an additional insult such as co-exposure to silica [38], which was the situation in our study. Interestingly, this study did not support our anecdotal suggestion, based on our earlier study [9], that higher Fe concentrations may be associated with a greater acute inflammatory response to geogenic PM_10_. This discrepancy can be reconciled by the fact that in the earlier study we only used two samples whereas in this study we used eight samples which allowed us to identify the specific contribution of Fe while controlling for the potential impact of other particle characteristics on the lung response.

Particle size has been demonstrated to impact on the respiratory response to inhaled particles. For example, there is an inverse association between particle size and the lung inflammatory response to silver nanoparticles [39] while there is a positive association for crystalline silica in the fine to ultrafine range [40]. Interestingly we found no association between adverse lung outcomes and MMAD; although it should be noted that variation in MMAD was not large (1.52–4.5 µm). We did, however, find that the *variability* in particle size (GSD) had a significant impact on both the inflammatory response and lung function deficit 7 days post-exposure. There are very few, if any, studies examining the impact of GSD on the lung response to inhaled particles. One explanation for our data is that a collection of particles with a wide size range is likely to initiate responses down multiple pathways. For example, a sample with a high GSD is likely to have a high mass weighted representation of fine and ultrafine particles which are known to *independently* have potent impacts on the lung inflammatory response; as compared to a sample with the same MMAD but a lower GSD. Notwithstanding the mechanism behind this association, this observation suggests that additional work is required to elucidate the impact of GSD on lung responses in community settings.

The concentration of Al was also associated with the magnitude of the “overshoot” in inflammatory markers below control levels we observed at the 7 day timepont. This response was associated with almost every physico-chemical characteristic we examined (except MMAD). Interestingly, a previous study comparing responses in human epithelial cell lines (BEAS-2B) to soil derived particulate matter of varying composition found the ratio of Al:Si to be a key determinant of the IL-6 response *in vitro* [4]. While we found no such association in our study (p = 0.46, *data not shown*), together these data support the notion that mineral heterogenity is an important determinant of the lung response to geogenic particulates.

It should be acknowledged that this study had some limitations. This study was exploratory and observational in nature, and therefore further work will be required to identify the causal pathways for the biological responses we observed. It should also be acknowledged that this study involved a single acute exposure to geogenic PM_10_ which may be unlike the typical situation of an individual in a community who is likely to be exposed chronically to these particles.

Notwithstanding these limitations, this study demonstrated that the lung response to a single dose of geogenic PM_10_ is complex and highly dependent on specific particle characteristics. We have identified that the concentration of Fe, while not being the only important characteristic, is strongly associated with many of the adverse lung responses to community sampled geogenic PM_10_.

## References

[1] Chow JC, Watson JG, Lowenthal DH (1993) PM10 and PM2.5 compositions in California’s San Joaquin Valley. Aerosol Sci Technol 18: 105–128.

[2] Quality of Urban Air Review Group (1996) Airborne particulate matter in the United Kingdom. Birmingham, UK: Department of Environment.

[3] Department of Environment and Conservation (2011) Western Australia air monitoring reports. Perth, Australia: Department of Environment and Conservation.

[4] Veranth JM, Moss TA, Chow JC, Labban R, Nichols WK, et al (2006) Correlation of *in vitro* cytokine responses with the chemical composition of soil-derived particulate matter. Environ Health Perspect 114: 341–349. 16507455 10.1289/ehp.8360 PMC1392226

[5] Hedenborg M, Klockars M (1989) Quartz dust induced production of reactive oxygen metabolites by human granulocytes. Lung 167: 23–32. 2537915 10.1007/BF02714927

[6] Doelman CJ, Leurs R, Oosterom WC, Bast A (1990) Mineral dust exposure and free radical-mediated lung damage. Exper Lung Res 16: 41–55. 2407528 10.3109/01902149009064698

[7] Mossman BT, Churg A (1998) Mechanisms in the pathogenesis of asbestosis and silicosis. Am J Respir Crit Care Med 157: 1666–1680. 9603153 10.1164/ajrccm.157.5.9707141

[8] Hnizdo E, Vallyathan V (2003) Chronic obstructive pulmonary disease due to occupational exposure to silica dust: a review of epidemiological and pathological evidence. Occupat Environ Med 60: 237–243. 10.1136/oem.60.4.237 PMC1740506 12660371

[9] Zosky GR, Boylen CE, Wong RS, Smirk MN, Gutierrez L, et al (2014) Variability and consistency in lung inflammatory responses to particles with a geogenic origin. Respirology 19: 58–66. 23796236 10.1111/resp.12150

[10] Andrews NC, Schmidt PJ (2007) Iron homeostasis. Ann Rev Physiol 69: 69–85. 17014365 10.1146/annurev.physiol.69.031905.164337

[11] Mossman BT, Borm PJ, Castranova V, Costa DL, Donaldson K, et al (2007) Mechanisms of action of inhaled fibers, particles and nanoparticles in lung and cardiovascular diseases. Particle Fibre Toxicol 4: 4. 10.1186/1743-8977-4-4 PMC1894816 17537262

[12] Zhou Y-M, Zhong C-Y, Kennedy IM, Leppert VJ, Pinkerton KE (2003) Oxidative stress and NFkB activation in the lungs of rats: a synergistic interaction between soot and iron particles. Toxicol Appl Pharmacol 190: 157–169. 12878045 10.1016/s0041-008x(03)00157-1

[13] Mullan N, Codde J, van Buynder P (2006) Respiratory hospitalisations in Port Hedland, 1993–2004: an exploratory geographical analysis. Perth, Australia: Department of Health.

[14] South Australian Department of Health (2007) Whyalla health impact study. Adelaide, Australia: Department of Health.

[15] Ljung K, Shan Siah W, Devine B, Maley F, Wensinger A, et al (2011) Extracting dust from soil: improved efficiency of a previously published process. Sci Total Environ 410: 269–270. 21907390 10.1016/j.scitotenv.2011.07.061

[16] Lakatos HF, Burgess HA, Thatcher TH, Redonnet MR, Hernady E, et al (2006) Oropharyngeal aspiration of a silica suspension produces a superior model of silicosis in the mouse when compared to intratracheal instillation. Exper Lung Res 32: 181–199. 16908446 10.1080/01902140600817465 PMC10208218

[17] Miller MA, Stabenow JM, Parvathareddy J, Wodowski AJ, Fabrizio TP, et al (2012) Visualization of murine intranasal dosing efficiency using luminescent *Francisella tularensis*: effect of instillation volume and form of anaesthesia. PLoS One 7: e31359. 22384012 10.1371/journal.pone.0031359 PMC3286442

[18] Adamson IYR, Prieditis H, Bowden DH (1993) Enhanced clearance of silica from moue lung after instillation of a leukocyte chemotactic factor. Exper Lung Res 20: 223–233. 10.3109/01902149409064384 7925140

[19] Boylen CE, Sly PD, Zosky GR, Larcombe AN (2011) Physiological and inflammatory responses in an anthropomorphically relevant model of acute diesel exhaust particle exposure are sex and dose dependent. Inhal Toxicol 23: 906–917. 22122304 10.3109/08958378.2011.625454

[20] Larcombe AN, Foong RE, Boylen CE, Zosky GR (2012) Acute diesel exhaust particle exposure increases viral titre and inflammation associated with existing influenza infection but does not exacerbate deficits in lung function. Influenza Other Respir Viruses. doi:10.1111/irv.12012. 10.1111/irv.12012 PMC5781203 22994877

[21] Zosky GR, Berry LJ, Elliot JG, James AL, Gorman S, et al (2011) Vitamin D deficiency causes deficits in lung function and alters lung structure. Am J Respir Crit Care Med 183: 1336–1343. 21297070 10.1164/rccm.201010-1596OC

[22] Janosi TZ, Adamicza A, Zosky GR, Asztalos T, Sly PD, et al (2006) Plethysmographic estimation of thoracic gas volume in apneic mice. J Appl Physiol 101: 454–459. 16645196 10.1152/japplphysiol.00011.2006

[23] Hantos Z, Daroczy B, Suki B, Nagy S, Fredberg JJ (1992) Input impedance and peripheral inhomogeneity of dog lungs. J Appl Physiol 72: 168–178. 1537711 10.1152/jappl.1992.72.1.168

[24] Fredberg JJ, Stamenovic D (1989) On the imperfect elasticity of lung tissue. J Appl Physiol 67: 2408–2419. 2606848 10.1152/jappl.1989.67.6.2408

[25] Zosky GR, Janosi TZ, Adamicza A, Bozanich EM, Cannizzaro V, et al (2008) The bimodal quasi-static and dynamic elastance of the murine lung. J Appl Physiol 105: 685–692. 18556435 10.1152/japplphysiol.90328.2008

[26] Hornung V, Bauernfeind F, Halle A, Samstad EO, Kono H, et al (2008) Silica crystals and aluminium salts activate the NALP3 inflammasome through phagosomal destabilization. Nature Immunol 9: 847–856. 18604214 10.1038/ni.1631 PMC2834784

[27] Kumar RK (1989) Quantitative immunohistologic assessment of lymphocyte populations in the pulmonary inflammatory response to intratracehal silica. Am J Pathol 135: 605–614. 2552810 PMC1880039

[28] Velan GM, Kumar RK, Cohen DD (1993) Pulmonary inflammation and fibrosis following subacute inhalational exposure to silica: determinants of progression. Pathology 25: 282–290. 8265248 10.3109/00313029309066590

[29] Cowie RL (1998) The influence of silicosis on deteriorating lung function in gold miners. Chest 113: 282–290. 10.1378/chest.113.2.340 9498949

[30] Moreas TJ, Zurawska JH, Downey GP (2006) Neutrophil granule contents in the pathogenesis of lung injury. Curr Opin Hematol 13: 21–27. 16319683 10.1097/01.moh.0000190113.31027.d5

[31] Grommes J, Soehnlein O (2011) Contribution of neutrophils to acute lung injury. Mol Med 17: 293–307. 21046059 10.2119/molmed.2010.00138 PMC3060975

[32] Venaille TJ, Ryan G, Robinson BW (1998) Epithelial cell damage is induced by neutrophil-derived, not pseudamonas-derived, proteases in cystic fibrosis sputum. Respir Med 92: 233–240. 9616518 10.1016/s0954-6111(98)90101-9

[33] Ohtsuka Y, Munakata M, Ukita H, Takahashi T, Satoh A, et al (1995) Increased susceptibility to silicosis and TNF-a production in C57BL/6 mice. Am J Respir Crit Care Med 152: 2144–2149. 8520788 10.1164/ajrccm.152.6.8520788

[34] Pasula R, Britigan BE, Turner J, Martin II WJ (2009) Airway delivery of silica increases susceptibility to mycobacterial infection in mice: potential role of repopulating macrophages. J Immunol 182: 7102–7109. 19454707 10.4049/jimmunol.0803642 PMC2748844

[35] Ratledge C, Dover LG (2000) Iron metabolism in pathogenic bacteria. Ann Rev Microbiol 54: 881–941. 11018148 10.1146/annurev.micro.54.1.881

[36] Carter JD, Ghio AJ, Samet JM, Devlin RB (1997) Cytokine production by human airway epithelial cells after exposure to an air pollution particle is metal-dependent. Toxicol Appl Pharmacol 146: 180–188. 9344885 10.1006/taap.1997.8254

[37] Aust AE, Ball JC, Hu AA, Lighty JS, Smith KR, et al (2002) Particle characteristics responsible for effects on human epithelial cells. Res Report Health Effects Inst 110: 1–76. 12578113

[38] Ghio AJ, Kennedy TP, Whorton AR, Crumbliss AL, Hatch GE, et al (1992) Role of surface complexed iron in oxidant generation and lung inflammation induced by silicates. Am J Physiol: Lung Cell Mol Physiol 263: L511–L518. 10.1152/ajplung.1992.263.5.L511 1332500

[39] Park MV, Neigh AM, Vermeulen JP, de la Fonteyne LJ, Verharen HW, et al (2011) The effect of particle size on the cytotoxicity, inflammation, developmental toxicity and genotoxicity of silver nanoparticles. Biomaterials 32: 9810–9817. 21944826 10.1016/j.biomaterials.2011.08.085

[40] Kajiwara T, Ogami A, Yamato H, Oyabu T, Morimoto Y, et al (2007) Effect of particle size of intratracheally instilled crystalline silica on pulmonary inflammation. J Occupat Health 49: 88–94. 10.1539/joh.49.88 17429165

